# Effect of capsular tension ring on the refractive outcomes of patients with extreme high axial myopia after phacoemulsification

**DOI:** 10.1186/s40001-024-01726-6

**Published:** 2024-02-24

**Authors:** Hui-Ying Zhao, Jing-Shang Zhang, Meng Li, Dong-Jun Chen, Xiu-Hua Wan

**Affiliations:** 1 Department of Ophthalmology, Beijing Geriatric Hospital, Beijing, China; 2 Beijing Tongren Eye Center, Beijing Tongren Hospital, Capital Medical University, Beijing Ophthalmology & Visual Sciences Key Laboratory, Beijing, China

**Keywords:** High axial myopia, Cataract, Capsular tension ring, Refraction, Intraocular lens power calculation formula

## Abstract

**Purpose:**

The aim of the study is to evaluate the effect of capsular tension ring (CTR) implantation following cataract surgery on the refractive outcomes of patients with extreme high axial myopia.

**Methods:**

Sixty eyes (with an axial length of ≥26 mm) were retrospectively reviewed and classified into two groups: CTR group (*n* = 30), which underwent CTR implantation following phacoemulsification, and control group (*n* = 30), which did not undergo CTR implantation. Intraocular lens (IOL) calculation was performed using Barrett Universal II (UII), Haigis, and SRK/T formulas. The refractive prediction error (PE) was calculated by subtracting the postoperative refraction from predicted refraction. The mean PE (MPE), mean absolute error (MAE), and percentages of eyes that had a PE of ±0.25, ±0.50, ±1.00, or ±2.00 diopters (D) were calculated and compared.

**Results:**

No significant differences were observed in PE between the two groups. The Barrett UII formula revealed a lower AE in the CTR group than in the control group (*p* = 0.015) and a lower AE than the other two formulas (p = 0.0000) in both groups. The Barrett UII formula achieved the highest percentage of eyes with a PE of ±0.25 D (66.67%).

**Conclusions:**

The refractive outcomes were more accurate in eyes with CTR implantation than in those with routine phacoemulsification based on the Barrett UII formula. The Barrett UII formula was recommended as the appropriate formula when planning CTR implantation in high myopia.

## Background

Capsular tension ring (CTR) is an important intraocular implantation device in cataract surgery. It is primarily implanted to stabilize the position of the capsular bag, facilitate phacoemulsification, and keep the intraocular lens (IOL) centered after surgery in eyes with zonular dehiscence and compromised capsular bag stability. Any cause of regional weakness or loss, such as pseudoexfoliation syndrome, trauma, previous ocular surgery (after vitrectomy), hypermature cataract, and increased axial length (AL) [1–4], may be an indication for CTR implantation. Patients with high myopia have longer zonular fibers because of the increased AL and thinner wall of the eyeball. Therefore, these patients are at increased risk of loosened capsular bag, unstable anterior chamber, and lens dislocation during cataract surgery [5]. The probability of capsular shrinkage is also higher after surgery [6]. Many clinicians have performed CTR implantation in patients with cataract and high myopia to increase the stability of IOL [5].

Currently, cataract surgery is considered a refractive surgery and not simply an extraction procedure. However, predicting refractive outcomes in eyes with increased AL remains challenging [7]. Studies regarding the effect of CTR implantation on the refractive outcomes in eyes with extreme high axial myopia are limited.

This study aimed to determine whether CTR implantation following cataract surgery can affect their refractive outcomes.

## Methods

### Patients

We retrospectively reviewed 60 patients with an axial eye length of ≥ 26 mm who had undergone phacoemulsification at the Department of Ophthalmology in our hospital between January and June 2023. Patients were classified into two groups: CTR group (*n* = 30), which underwent CTR implantation following phacoemulsification, and control group (*n* = 30), which did not undergo CTR implantation. The CTR group was matched with the control group in terms of age and sex. Patients were included in this study if they were aged 18–90 years. Exclusion criteria were as follows: (1) eyes that underwent ultrasound biometry rather than optical biometry using a partial coherence interferometry device (IOLMaster, Carl Zeiss Meditec) in cases of dense cataract; (2) eyes that underwent previous ocular or intraocular surgery and showed evidence of trauma, corneal infection, or postoperative complications. Only one eye per patient was selected for evaluation. If both eyes of the same patient fulfilled the abovementioned criteria, then the eye was randomly selected for examination.

Written informed consent was obtained from all patients for analysis and publication of their anonymized clinical data.

### Surgical technique

All surgeries were performed under local anesthesia. All enrolled patients underwent phacoemulsification and IOL implantation (PCF60/A, Henan universe intraocular lens research company, China). A 3.0-mm corneal incision was made, 5.5–6.0 mm continuous curvilinear capsulorhexis was performed, and monofocal acrylic IOL was implanted. The CTR group underwent CTR implantation (ACPi-11, Bausch & Lomb, Rochester, NY, USA) before IOL implantation. All surgical procedures were performed by an experienced surgeon (Xiu-hua Wan).

### Data collection

In all cases, preoperative biometry was performed using a partial coherence interferometry device (IOLMaster700, Zeiss, Oberkochen, Germany). Keratometry values, white-to-white (WTW) diameter, anterior chamber depth (ACD), lens thickness (LT), and ALs were reviewed. In addition, IOL calculation was performed via IOLMaster700 software using Barrett UII, Haigis, and SRK/T formulas.

**Fig. 1:**
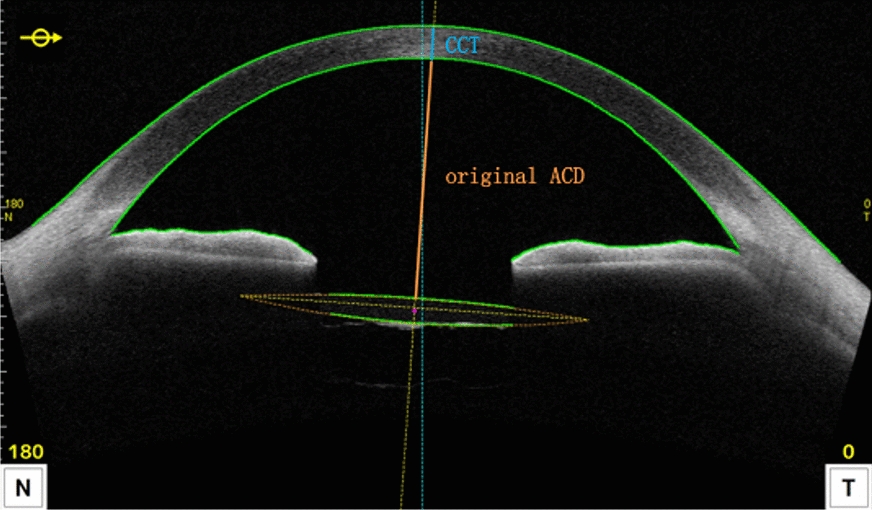
A representative image with anterior segment parameters of the CASIA2. *CCT* central corneal thickness, *ACD* anterior chamber depth

Refraction measurements and postoperative ACD were obtained 3 months postoperatively. Postoperative ACD in pseudophakic eyes was measured using CASIA2 (AS-OCT, CASIA2, Tomey Corp.). The original ACD, which was measured using the CASIA2 system, refers to the distance from the corneal endothelium to the anterior surface of IOL. The postoperative ACD value was calculated as the sum of the original ACD and central corneal depth (Fig. 1).

To measure the accuracy and variance of the refractive outcomes, the refractive prediction error (PE) was calculated by subtracting the postoperative refraction from predicted refraction. A negative PE value represents a hyperopic shift from the predicted refraction, whereas a positive PE value represents a myopic shift from the predicted refraction. The absolute error (AE) indicates the absolute value of PE. In addition, the mean PE (MPE), mean absolute PE (MAE), median AE (MedAE), and the number and percentages of eyes within a PE of ± 0.25, ± 0.50, ± 1.00, and ± 2.00 diopters (D) were calculated using each formula.

The change in the ACD value was calculated by subtracting the preoperative ACD value from the postoperative ACD value.

### Statistical analysis

Statistical analysis was performed using SPSS Statistics software (v. 18, SPSS Inc.). Independent *t* test was performed to compare keratometry values, WTW diameter, ACD, LT, AL, PE, AE, and change in ACD between the two groups. The differences in PEs, AEs obtained via different formulas were assessed using paired-samples *t* test. A *p* value of < 0.05 was considered to indicate statistical significance.

## Results

### Patient characteristics

Table 1 shows the preoperative data of patients in both groups. No statistically significant difference was observed in the corneal power, AL, WTW diameter, ACD, and LT between the two groups.

**Table 1:** Preoperative biometric data

Parameter	CTR group (*n* = 30)		Control group (*n* = 30)	
	Mean	SD	Mean	SD
K (D)	43.65	1.29	44.16	1.63
AL (mm)	30.09	2.07	30.18	2.12
WTW diameter (mm)	11.69	0.46	11.65	0.52
ACD (mm)	3.42	0.42	3.44	0.38
LT (mm)	4.42	0.51	4.49	0.28

*CTR* capsular tension ring, *K* keratometry, *AL* axial length, *WTW* white-to-white, *ACD* anterior chamber depth, *LT* lens thickness

### MPE and MAE

Table 2 shows the MPE and MAE values in both CTR and control groups. Our findings revealed no statistically significant differences in MPE between the two groups using all three formulas. In the CTR group, Barrett UII, Haigis, and SRK/T formulas revealed negative PE values in 14 (46.7%), 28 (93.3%), and 24 (80%) eyes, respectively. In contrast, in control group, the three formulas showed negative PE values in 12 (40%), 24 (80%), and 23 (76.7%) eyes, respectively.

**Table 2:** The mean arithmetic and mean absolute refractive prediction errors (D) using the Barrett Universal II (UII), Haigis, and SRK/T formulas

Parameter	Formula	Mean	SD	Range	Median (25–75%)
CTR group					
PE	Barrett UII	0.06	0.39	− 1.07 to 1.05	0.05 (− 0.15 to − 0.2)
	Haigis	− 0.34	0.42	− 1.64 to 0.75	− 0.36 (− 0.58 to − 0.09)
	SRK/T	− 0.31	0.46	− 1.5 to 0.7	− 0.37 (− 0.55 to − 0.03)
AE	Barrett UII	0.28	0.27	0.01 to 1.07	0.17 (0.09 to 0.29)
	Haigis	0.41	0.35	0.01 to 1.64	0.41 (0.12 to 0.59)
	SRK/T	0.43	0.34	0.01 to 1.5	0.44 (0.15 to 0.55)
Control group					
PE	Barrett UII	0.12	0.67	− 2.03 to 1.29	0.18 (− 0.2 to 0.5)
	Haigis	− 0.40	0.72	− 2.58 to 0.91	− 0.34 (− 0.87 to − 0.07)
	SRK/T	− 0.38	0.82	− 2.75 to 1.54	− 0.37 (− 0.78 to − 0.07)
AE	Barrett UII	0.51	0.43	0.02 to 2.03	0.41 (0.2 to 0.68)
	Haigis	0.58	0.57	0.02 to 2.58	0.42 (0.07 to 0.83)
	SRK/T	0.62	0.64	0.03 to 2.75	0.44 (0.16 to 0.83)

Based on the Barrett UII formula, MAE in the CTR group was lower than that in the control group (*t* = − 2.533, *p* = 0.015). No statistically significant difference was noted in MAE between the two groups using the Haigis (*t* = − 1.336, *p* = 0.188) or SRK/T (*t* =  − 1.465, *p* = 0.15) formula. However, a lower variance (i.e., higher precision) in  AE was observed in the CTR group using Haigis and SRK/T formulas (*p* = 0.036 and *p* = 0.007, respectively; Levene test).

In the CTR group, the Barrett UII formula revealed a significantly lower MAE than Haigis (*t* = 5.846, *p* = 0.0000) and SRK/T (*t* = 4.505, p = 0.0000) formulas. Further, the Barrett UII formula revealed a lower MAE than Haigis (*t* = 5.067, *p* = 0.0000) and SRK/T (*t* = 3.921, *p* = 0.0000) formulas in the control group.

**Fig. 2:**
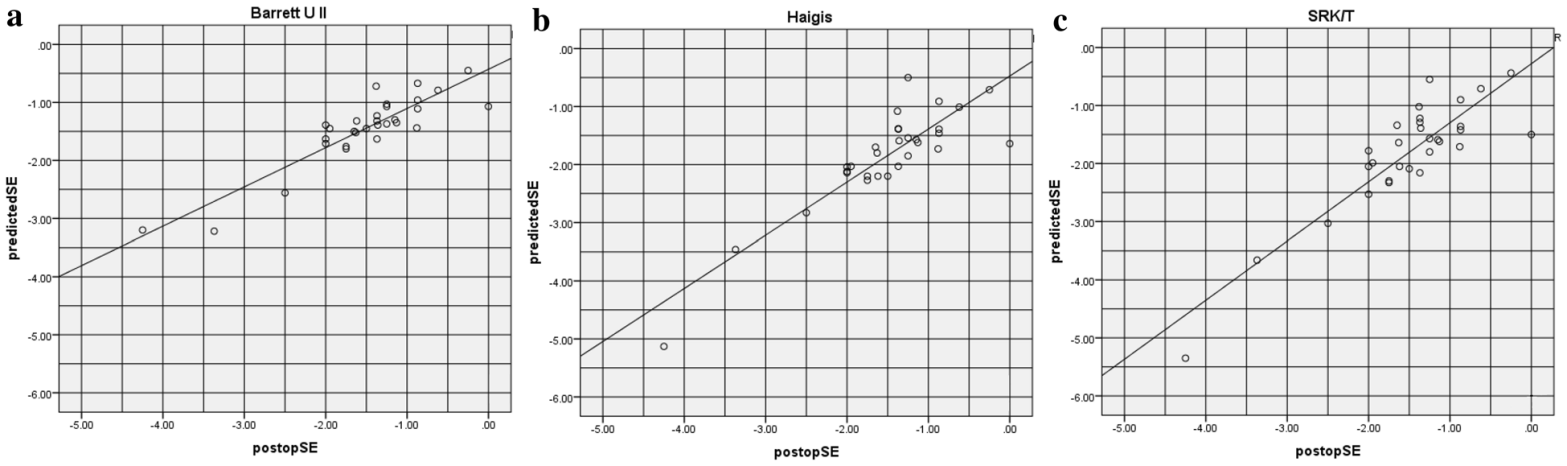
Predicted SE refraction value obtained using Barrett UII (**a**), Haigis (**b**), and SRK/T (**c**) formulas versus the postoperative SE value in the CTR group (*SE* spherical equivalent)

**Fig. 3:**
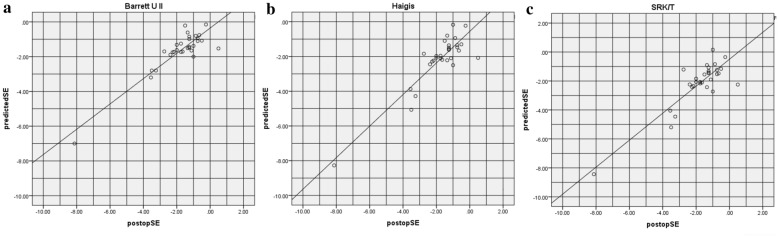
Predicted SE refraction using the Barrett UII (**a**), Haigis (**b**), and SRK/T (**c**) formulas versus the postoperative SE in the control group (*SE* spherical equivalent)

Figures 2 and 3 show the predicted spherical equivalent (SE) refraction versus postoperative SE refraction in both groups. A hyperopic shift (shift of the linear to the right) can be observed in Figs. 2b, c and 3b, c. A myopic shift (shift of the linear to the left) can be observed in Fig. 3a.

### Percentages of eyes within PEs of ± 0.25, ± 0.50, ± 1.00, or ± 2.00 D

**Fig. 4:**
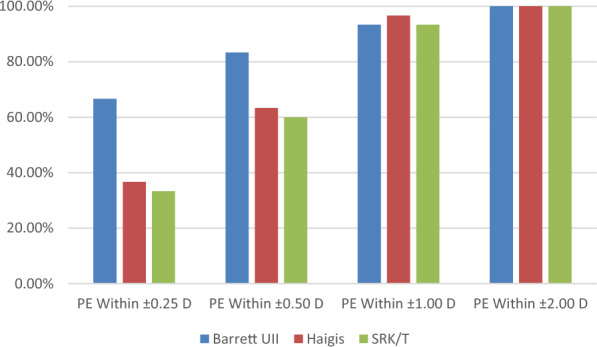
Percentages of eyes within a certain refractive prediction error (PE) in the CTR group

**Fig. 5:**
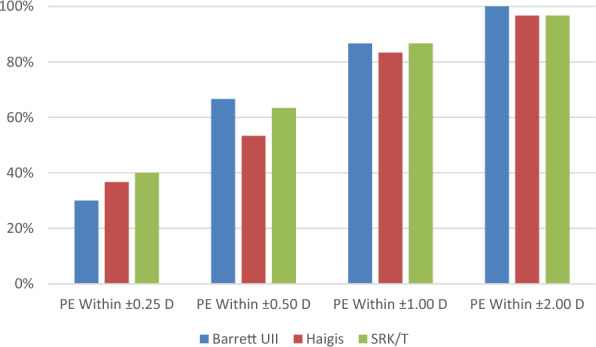
Percentages of eyes within a certain refractive prediction error (PE) in the control group

Figures 4 and 5 show the percentages of eyes within a PE of ± 0.25, ± 0.50, ± 1.00, or ± 2.00 D in both groups. In the CTR group, the percentage of eyes within a PE of ± 0.25 D was 66.67% (20/30) based on the Barrett UII formula, which was higher than that obtained via the other two formulas. Further, the percentage of eyes within a PE of ± 0.50 D based on the Barrett UII formula was 83.33% (25/30), which was higher than that obtained via Haigis (63.33%, 19/30) and SRK/T (60%, 18/30) formulas.

### Postoperative ACD and change of ACD values

The mean postoperative ACD values were 5.06 ± 0.39 (range 4.07–5.92) and 5.04 ± 0.34 (range 4.50–5.74) mm as well as mean changes in ACD values were 1.64 ± 0.32 (range 0.95–2.25) and 1.59 ± 0.35 (range 0.84–2.32) mm in CTR and control groups, respectively. There were no significant differences in change of ACD values between the two groups (*p* = 0.623).

## Discussion

It remains controversial whether CTR implantation during cataract surgery affects postoperative refractive PE. A retrospective case–control study compared 29 eyes with CTRs with 29 eyes without CTRs according to the SRK/T formula and revealed no statistically significant difference in MPE or MAE [8]. Another retrospective case–control series of 19 eyes with CTR implantation showed no statistically significant difference in the refractive PE values between CTR and control groups [9]. Consequently, the authors suggest that CTR implantation does not consistently affect refractive outcomes compared with routine phacoemulsification, and intraocular lens power can be calculated as usual when CTRs are used [8, 9]. This finding is consistent with that of other studies [5, 10]. However, Belov et al. compared 18 and 19 eyes with and without CTR coimplantation, respectively, and revealed a higher hyperopic IOL power calculation error of 0.41 ± 0.52 D in eyes with CTR coimplantation than in the control group (0.04 ± 0.59 D; *p* = 0.043) [11]. The result indicated that patients with weak zonules who underwent CTR coimplantation showed higher number of hyperopic IOL power calculation errors than those in the control group [11]. Another study on CTR implantation in 25 patients with abnormal zonules revealed a posterior shift of posterior chamber IOL after CTR implantation, leading to the requirement of hypermetropic correction after surgery. Thus, it was suggested that implanted IOL should exceed the preoperatively calculated value by + 1.0Dto +2.0 D when combined with CTR [12].

Studies regarding the effect of CTR implantation on the refractive outcomes in eyes with high axial myopia are limited. Schild et al. assessed the refractive outcomes in 31 myopic eyes (with an AL exceeding 25.5 mm) with (*n* = 16 eyes) or without (*n* = 15 eyes) CTR implantation and reported no statistically significant difference in MPE and MAE between the CTR and control groups using Haigis and SRK/T formulas [13]. Thus, this previous study indicated that CTR implantation had no consistent effect on refractive outcomes compared with routine phacoemulsification in highly myopic eyes, and no change in IOL power calculation is required [13]. Moreover, Yang et al. compared the PEs in myopic eyes with (*n* = 16 eyes) or without (*n* = 15 eyes) CTR implantation and revealed no significant differences [5].

Importantly, we revealed whether CTR implantation during phacoemulsification affects refractive outcomes in highly myopic eyes (AL of ≥  26 mm) depending on the IOL calculation formulas used. Similar to a previous study [13], we revealed no statistically significant difference in MPE or MAE between the CTR and control groups using Haigis and SRK/T formulas. A lower variance was observed in the MAE of the CTR group using Haigis and SRK/T formulas, which was identical to that in the previous study [13]. Thus, we indicated that CTR implantation had no significant effect on the refractive outcomes of patients with high axial myopia after phacoemulsification based on the Haigis or SRK/T formula and revealed a tendency toward higher precision in outcomes with CTR implantation.

However, we revealed that the refractive outcomes were more accurate in eyes with CTR implantation when the IOL calculation was performed using the Barrett UII formula. To the best of our knowledge, this is the first study to compare the refractive outcomes in highly myopic eyes (AL of ≥  26 mm) with or without CTR coimplantation. This study revealed that the Barrett UII formula showed a lower MAE and higher percentage of eyes within PEs of ± 0.25 D in the CTR group (0.28 ± 0.27 D, 66.67%) than in the control group (0.51 ± 0.43 D, 30%).

We speculate that the difference in conclusion concerning the refractive outcomes may derive from the discrepant accuracy of the formulas. It is difficult to predict refractive outcomes in eyes with increased AL [7]. When selecting IOLs for high and extreme myopia, the selection of appropriate formulas can yield accurate refractive outcomes that meet the standard criteria [14]. The Barrett UII formula was believed to be a more accurate predictor of actual postoperative refraction than the other formulas in eyes with an AL of > 26 mm [7]. Kane et al. assessed the AEs among the seven IOL power formulas, including Haigis and SRK/T formulas, in eyes with increased AL (> 26 mm; *n* = 77 eyes) and found that the Barrett UII formula showed the lowest MAE and the highest percentage (42.7%) of eyes within a PE of ± 0.25 D among all formulas. The SRK/T formula showed lower AEs than all other formulas, except for the Barrett UII formula [7]. Similarly, the Barrett UII formula revealed the lowest MAE and highest percentage of eyes within a PE of ± 0.5 D in both groups in this study. In addition, we revealed that the Barrett UII formula was the most accurate formula among the three formulas in highly myopic eyes with CTR implantation. This study revealed that the Barrett UII formula demonstrated the lowest MAE and highest percentage of eyes within PEs of ± 0.25 D in the CTR group among the three formulas. Thus, the Barrett UII formula was recommended as the appropriate formula in cases of high myopia, regardless of CTR implantation. Therefore, we believe that the refractive outcomes were more accurate in eyes with CTR implantation.

Based on these results, CTR implantation may induce refractive outcomes more accurately by affecting the IOL location. The CTR was designed to stretch the lens capsule and uniformly balance the tension of zonular fibers, thereby improving the stability of IOL [15]. Our study revealed that the change in ACD values of eyes with CTR was 0.05 mm, which was larger than that in eyes without CTR. This finding was consistent with refractive outcomes obtained using the Barrett UII formula, which revealed that MPE decreased by 0.06 D in cases of CTR implantation compared with that in cases without CTR implantation. However, the difference in the change in ACD values between CTR group and control group was not significant. Interestingly, it remains controversial whether CTR implantation during cataract surgery affects the postoperative axial intraocular lens position. A case–control study assessed postoperative ACD in 60 eyes and revealed no difference in the change in ACD value between CTR and control groups [16]. A study by Yang et al. revealed that the depth of the central anterior chamber did not differ significantly between the CTR and control groups in eyes with a history of PPV or severe myopia postoperatively [5]. However, Baranwal et al. assessed the ACD of 25 patients with CTR and revealed a 0.15–0.25-mm posterior shift of IOL [12]. Thus, our hypothesis should be validated in further studies.

This study has certain limitations. First, the present study was retrospective and had a small sample size. Second, although individualized optimization of the CTR diameter is more desirable for patients with high myopia, CTR with a diameter of 11 mm was used for all patients. Finally, there is possible selection bias as our follow-up period was only 3 months after surgery.

## Conclusions

The effects of CTR implantation during phacoemulsification on the refractive outcomes in extremely high axial myopic eyes (AL of  ≥ 26 mm) varied depending on the IOL calculation formulas used. The Barrett UII formula was the most accurate formula among the three formulas in high axial myopic eyes, regardless of CTR implantation. The refractive outcomes were more accurate in eyes with CTR implantation based on the Barrett UII formula.

## Data Availability

The datasets used and/or analyzed during the current study are available from the corresponding author on reasonable request.

## Abbreviations

ACD: Anterior chamber depth
AE: Absolute error
AL: Axial length
CTR: Capsular tension ring
IOL: Intraocular lens
LT: Lens thickness
MAE: Mean absolute error
MedAE: Median absolute error
MPE: Mean prediction error
PE: Prediction error
WTW: White-to-white
